# Co-culture with Endothelial Progenitor Cells promotes the Osteogenesis of Bone Mesenchymal Stem Cells via the VEGF-YAP axis in high-glucose environments

**DOI:** 10.7150/ijms.52316

**Published:** 2021-02-04

**Authors:** Peilian Wu, Xia Zhang, Yun Hu, Dongrong Liu, Jinlin Song, Wenjie Xu, Hao Tan, Rui Lu, Leilei Zheng

**Affiliations:** 1 The Affiliated Stomatology Hospital, Chongqing Medical University, Chongqing, 401147, China.; 2 Chongqing Key Laboratory of Oral Diseases and Biomedical Sciences, Chongqing, 401147, China.; 3 Chongqing Municipal Key Laboratory of Oral Biomedical Engineering of Higher Education, Chongqing, 401147, China.; 4 West china dental hospital of Chongqing, Chongqing, 401147, China.

**Keywords:** type 2 diabetes mellitus, High glucose, BMSCs, EPCs, VEGF, YAP

## Abstract

Patients with type 2 diabetes mellitus (T2DM) have a high risk of fracture and experience poor bone healing. In recent years, bone mesenchymal stem cells (BMSCs) and endothelial progenitor cells (EPCs) have become the most commonly used cells in cell therapy and tissue engineering. In this study, we found that high glucose levels had a negative effect on the differentiation of BMSCs and EPCs. Considering that EPCs-BMSCs sheets can provide endothelial cells and osteoblastic cells, we transplanted cell sheets into T2DM rats with bilateral skull defects. The outcomes of the *in vivo* study revealed that EPCs-BMSCs sheets promoted ossification, which was verified by micro-CT and immunohistochemistry (IHC) analyses. Furthermore, we detected the VEGF content in the culture supernatant using an enzyme-linked immunosorbent assay (ELISA). The results showed that the BMSCs co-cultured with EPCs presented a higher level of VEGF than other cells. To assess the differentiation and migration of BMSCs exposed to VEGF, ALP staining, scratch assay and qRT-PCR analysis were performed. In addition, we used immunofluorescence and western blotting analysis to further explore the related mechanisms. The results showed that cells cultured with VEGF had a stronger actin cytoskeleton and a greater amount of nuclear and total YAP than cells cultured without VEGF. Taken together, our results indicate that co-culture with EPCs could promote the osteogenesis of BMSCs partially via VEGF. Furthermore, YAP and F-actin play important roles in this process.

## Introduction

Diabetes mellitus (DM) is a worldwide health problem with increasing prevalence 1, and approximately 90% of patients with diabetes suffer from type 2 diabetes mellitus (T2DM) 2. Common complications of diabetes include increased fracture risk and impaired bone quality, which are related to hyperglycaemia 3, 4. Specifically, DM not only impairs bone repair but also limits the capacity of neovascularization 5. Considering the high incidence of diabetes and its considerable negative effect on bone healing, the aim of this article is to provide new ideas for better treatment strategies.

Bone marrow mesenchymal stem cells (BMSCs) are multi-lineage cells with self-renewal and differentiation capabilities and are the most commonly used stem cells in cell therapy and tissue engineering 6. In recent years, BMSCs have become the main source of osteoblasts and an important part of bone healing and regeneration. Insufficient vascularization is one of the challenges in repairing large bone defects in tissue engineering. Endothelial progenitor cells (EPCs), as ideal angiogenic cells, have become the focus of tissue engineering research 7. Additionally, they can promote angiogenesis by producing pro-angiogenic factors or can differentiate into endothelial cells and integrate into newly formed blood vessels 8. These properties make EPCs attractive candidates for stem cell therapy. Recent investigations have revealed that the differentiation of stem cells is strongly associated with glucose metabolism 4, 9. It is of interest to test whether high glucose levels affect the differentiation of BMSCs and EPCs.

Bone marrow-derived EPCs and MSCs have the same origin and simultaneously exist in bones and wound healing sites where vascular regeneration is required, and direct contact between these two cell types can be established at these sites 10. Some studies have shown that EPCs can provide a local environment conducive to the osteogenic differentiation of BMSCs, and the combination of BMSCs and EPCs can promote osteoblast differentiation *in vitro* and promote bone healing *in vivo* by supporting bone regeneration 11. Nevertheless, it remains unclear whether EPCs could be combined with BMSCs to improve bone healing in patients with diabetes. The mechanisms underlying the effect of co-culturing BMSCs with EPCs are, however, still not completely understood.

The proliferation and recruitment of MSCs and their differentiation into mature osteoblasts are regulated by a variety of factors, including growth factors, cytokines, and others. These factors are released by the osteoblasts themselves to some extent and by cells that are part of the tightly connected vascular system, such as endothelial cells 12. Studies have shown that the proliferation and activity of osteoblast-associated cells decrease and that the reduction in the expression of key growth factors, such as vascular endothelial growth factor (VEGF), is the main reason for the negative effects of diabetes on bone formation and bone defects. VEGF plays a key role not only in mediating angiogenesis responses in multiple physiological and pathological processes 13 but also in bone healing 14. Therefore, we wondered whether VEGF can promote BMSC osteogenesis under high-glucose conditions and explored the potential mechanisms.

## Materials and Methods

### Animals

Male Sprague-Dawley rats (6 weeks of age) were purchased from the Experimental Animal Center of Chongqing Medical University (Chongqing, China) and raised under stabilized conditions at a humidity of 48% and a temperature of 20 °C. All animal experiments were conducted in accordance with the regulations of the Ethics Committee of the International Association for the Study of Pain and the guidelines of the Bioethics Committee for Animal Research of Chongqing Medical University.

### Cell isolation and culture

Rat BMSCs and EPCs were isolated as described in previous articles 11, 15. BMSCs were cultured in Dulbecco's modified Eagle's medium (DMEM, HyClone, USA) containing 10% foetal bovine serum in a humidified incubator at 37 °C with 5% CO2. Third-passage (P3) or fourth-passage (P4) cells were used for subsequent experiments. The EPCs were cultured in M199 medium (HyClone, USA) containing 20% foetal bovine serum, 10 μg/L VEGF, 4 μg/L bFGF and 1% penicillin in a humidified incubator. Primary (P0) or first-passage (P1) cells were used for subsequent experiments.

### Differentiation and identification of BMSCs and EPCs

To detect the multi-directional differentiation ability of BMSCs, cells were assessed with Oil Red O solution, Alcian stain, alizarin red and cell surface marker antibodies as reported 16. Capillary tube formation, uptake of DiI-conjugated acetylated LDL (DiI-Ac-LDL) and fluorescin isothiocyanate-labelled *Ulex europaeus* agglutinin 1 (FITC-UEA-1) (Sigma, USA, all), and cell surface marker expression were assessed in EPCs. All experiments were performed according to the instructions. Tubule formation was observed under an inverted phase-contrast microscope (Nikon, Japan), and the staining results were observed under a fluorescence microscope.

### Cell proliferation assays

The Cell Counting Kit-8 (CCK-8; Tongren, Japan) assay was applied to assess BMSC or EPC viability according to the manufacturer's protocol. The cells were cultured in media containing 5.5- or 30-mM glucose for 7 days after being cultured routinely for 24 h. To indicate the number of living cells, the optical density was measured at 490 nm after further incubation for 2 h at 37 °C.

### Alkaline phosphatase (ALP) activity and staining

BMSCs were induced in osteogenic media for 4 or 7 days after being cultured routinely for 24 h. ALP enzymatic activity was analysed using an ALP detection kit, and ALP staining was assessed using an alkaline phosphatase staining kit (Nanjing Jiancheng Bioengineering Research Institute, China, all) according to the manufacturer′s instructions.

### Quantitative real-time polymerase chain reaction (qRT-PCR)

qRT-PCR analysis was performed according to previously described methods 17. Total RNA was isolated from cells using TRIzol reagent (Invitrogen, USA) following the instructions. The PrimeScript™ RT reagent Kit (TaKaRa, Japan) was used to convert total RNA into cDNA. Real-time PCRs were performed using the SYBR® Premix DimerEraser™ Kit (TaKaRa) and the Bio-Rad IQ5 Real-Time PCR Detection System (Bio-Rad). The primers used are listed in Table 1.

### Protein isolation and western blot analysis

Total proteins were extracted from the cells by lysis in RIPA buffer. The protein concentration in the extracted lysates was determined using a bicinchoninic acid (BCA) protein concentration assay kit (Beyotime Biotechnology, China). The cell lysates were separated by 10% SDS-PAGE and then transferred to a polyvinylidene fluoride (PVDF, Millipore, USA) membrane. After the transfer was completed, the membranes were blocked with 5% nonfat milk for 2 h. Then, the membranes were incubated at 4 °C with different primary antibodies overnight. Later, the membranes were incubated with the secondary antibody HRP for 2 h at room temperature. Finally, the bands were developed using enhanced chemiluminescence reagent (Thermo Scientific). Analysis of the relative density of the bands for immune response-related proteins was performed with ImageJ software. The antibodies used were as follows: VEGF (1:1000) and β-actin (1:1000) from Abcam and YAP (1:1000) and GAPDH (1:1000) from Cell Signaling Technology.

### Scratch assay

BMSCs were cultured on six-well plates until the cells reached 90% confluence and were then serum starved for 6 h and treated with VEGF. A wound was made with a pipette tip by scraping the cell monolayer. Lines were drawn on the backs of the six-well plates as nearby reference points. Images of the cell scratches were taken at 0 and 24 h, and a microscope was used to record the width of each scratch. The experiments were repeated three times, and a representative photograph was chosen.

### Enzyme-linked immunosorbent assay (ELISA)

BMSCs and EPCs were co-cultured in direct contact at a 1:1 ratio or were cultured alone; for this, were seeded onto 24-well plates in conditioned medium. The cell culture supernatants were assessed by a rat VEGF ELISA kit (ab100786; Abcam, Japan) according to the instructions.

### Immunofluorescence staining

BMSCs were cultured on 24-well plates with cover glasses. After treatment with or without VEGF for 24 h, cells were fixed with 4% paraformaldehyde for 15 min and treated with 0.2% Triton X-100. Some cells were incubated with phalloidin (Molecular Probes) solution for 30 min, and the nuclei were labelled with 4,-6-diamidino-2-phenylindole (DAPI, Invitrogen, USA). Others were blocked with 5% goat serum for 30 min and incubated with YAP rabbit mAb (Cell Signaling Technology, USA) overnight at 4 ℃. Afterwards, the cells were incubated with Alexa Fluor 488-conjugated goat anti-rabbit antibody (Bioss, China) for 90 min at room temperature. Finally, the nuclei were labelled with DAPI. The cells on the slides were visualized by a spectral confocal laser-scanning microscope (LSCM, Germany).

### Construction and observation of cell sheets

Cell sheet formation was performed according to a previously described method 18. Cells were seeded in 6-cm petri dishes, and the medium was shifted to cell sheet-inducing medium after the cells reached 90% confluence. After 14 days, cell sheets had formed. The cell sheets were fixed with 2.5% glutaraldehyde and then dehydrated with ethanol. The cell sheets were examined by a scanning electron microscope (Hitachi, Japan) after they were coated with gold.

### Animal experiments

#### Establishment of bilateral skull defects in T2DM rats

We established a diabetic rat model by intraperitoneally injecting rates with streptozotocin and feeding them a high-glucose/high-fat diet, as described in previous studies 19. After construction of the rat model of T2DM, general anaesthesia with 10% chloral hydrate was administered to rats by intraabdominal injection, and primacaine was injected into the operation area for local infiltration anaesthesia. The bilateral skull defects were located between the bilateral coronal and herringbone sutures and separated by the sagittal suture as the midline. Then, we generated bilateral skull bone defects of 4 mm in diameter with dental grinders.

### Experimental groups

The T2DM model rats were randomly divided into 4 groups of 3 rats each: (A) the blank control group; (B) the EPCs sheet group; (C) the BMSCs sheet group; and (D) the BMSCs/EPCs composite sheet group. Nothing was implanted in the skull defects of the rats in group A, which acts as a blank control. EPCs sheets were implanted in the skull defects of group B rats, BMSCs sheets were implanted in the skull defects of group C rats, and BMSCs/EPCs composite sheets were implanted into the skull defects of group D. The wounds were sutured in layers, and penicillin was injected to prevent infection for 7 days.

### Sample preparation

The animals were sacrificed at 8 weeks after sheets were implanted. Skull tissue 1 cm from the centre of the healing area was collected with a small dental sander. All samples were fixed in 4% paraformaldehyde solution for the following analyses:

#### Micro-CT analysis

Scanning of skull defect samples was performed using a micro-CT system (Viva CT40, SCANCO, Switzerland). A greyscale value from 700 to 2000 represents new bone. 3D models were reconstructed from micro-CT scan data sets for quantitative analysis of bone formation. Analyses of factors including the percentage of bone volume (BV/TV), trabecular number (Tb.N), trabecular thickness (Tb.Th), and trabecular separation (Tb.Sp) were performed. The region of interest (ROI) selected for the following analysis was defined as a cylinder with a diameter of 5 mm and a thickness of 1 mm.

#### Immunohistochemistry (IHC)

Skull tissue sections were fixed with 4% paraformaldehyde, dehydrated, and embedded, and IHC was performed after sectioning according to standard protocols. Paraffin sections were incubated with OCN or VEGF (1:200, both from Abcam, UK) followed by deparaffinization, microwave antigen retrieval and blocking of nonspecific immunoreactions. Sections were then incubated with biotinylated goat anti-rabbit IgG. Immunoreactivity was detected using a DAB complex kit (ZSJQB, China). Finally, the film was sealed and observed using a digital 3-camera microscope (Motic, China).

### Statistical analysis

Statistical analysis was performed using GraphPad Prism, and all data are shown as the mean ± SD. Data were analysed by Student's t-test or one-way ANOVA followed by Tukey's post-test, and a value of p<0.05 was considered statistically significant.

## Results

### Characterization of BMSCs and EPCs

**Figure 1:**
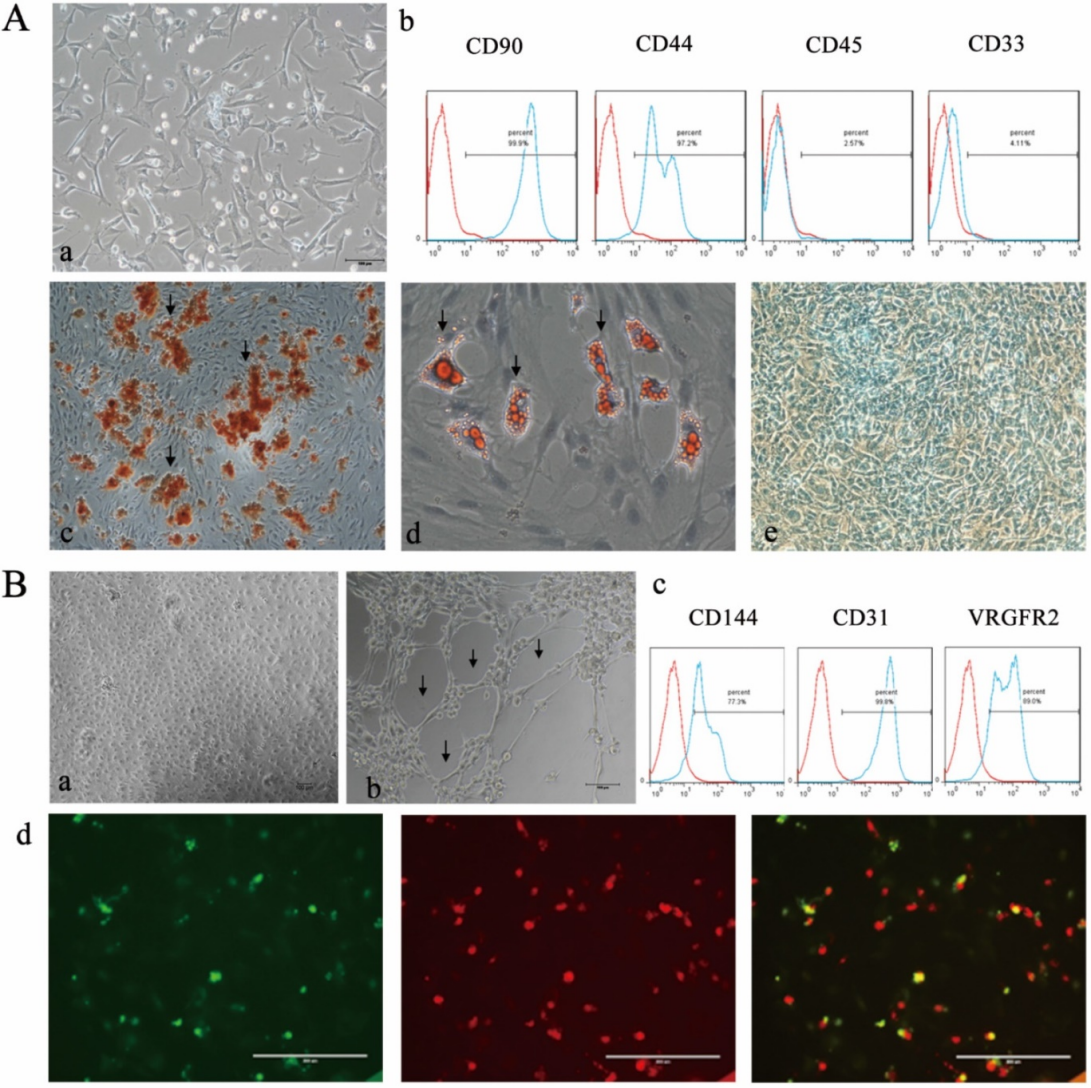
** Characterization of BMSCs and EPCs. A)** Isolation and differentiate of BMSCs a morphology of BNSCs,P0×100; **b)** Cell surface markers of BMSCs **c)** Alizarin red staining of BMSCs after osteogenic induction (×100; Black arrows indicate calcium deposits); **d)** Oil red O staining of BMSCs after adipogenic induction (×200; The black arrow indicates lipid droplets); e Alcain staining of BMSCs after chrondrogenic induction (×100). **B)** Isolation and identification EPCs **a)** morphology of epc, P1×100; **b)** Matrigel tubule formation experiment of EPCs (×100.Black arrow indicates tubule). **c)** Cell surface markers of EPCs **d)** Double fluorescence staining experiment of EPCs, (×200.green: FITC-UEA-1 red: DiI-Ac-LDL, yellow: fluorescence coincidence).

The isolated BMSCs maintained a spindle-shaped morphology (Figure 1Aa), expressed CD90 and CD44 and were negative for CD45 and CD33 (Figure 1Ab). Alizarin Red staining showed red mineralized nodules (Figure 1Ac). In addition, the adipogenic and chondrogenic differentiation abilities of BMSCs were detected by positive Oil Red O and Alcian staining (Figure 1Ad and Ae). EPCs formed small clusters and exhibited a “paving stone”-shaped morphology (Figure 1Ba) and expressed CD144, CD31 and VEGFR2 (Figure 1Bc). The tubule formation assay demonstrated the angiogenic potential of EPCs. (Figure 1Bb). Double-positive fluorescence staining for DiI-Ac-LDL and FITC-UEA-I confirmed the endothelial phenotype of EPCs (Figure Bd).

### Effects of high glucose on BMSC proliferation and differentiation

**Figure 2:**
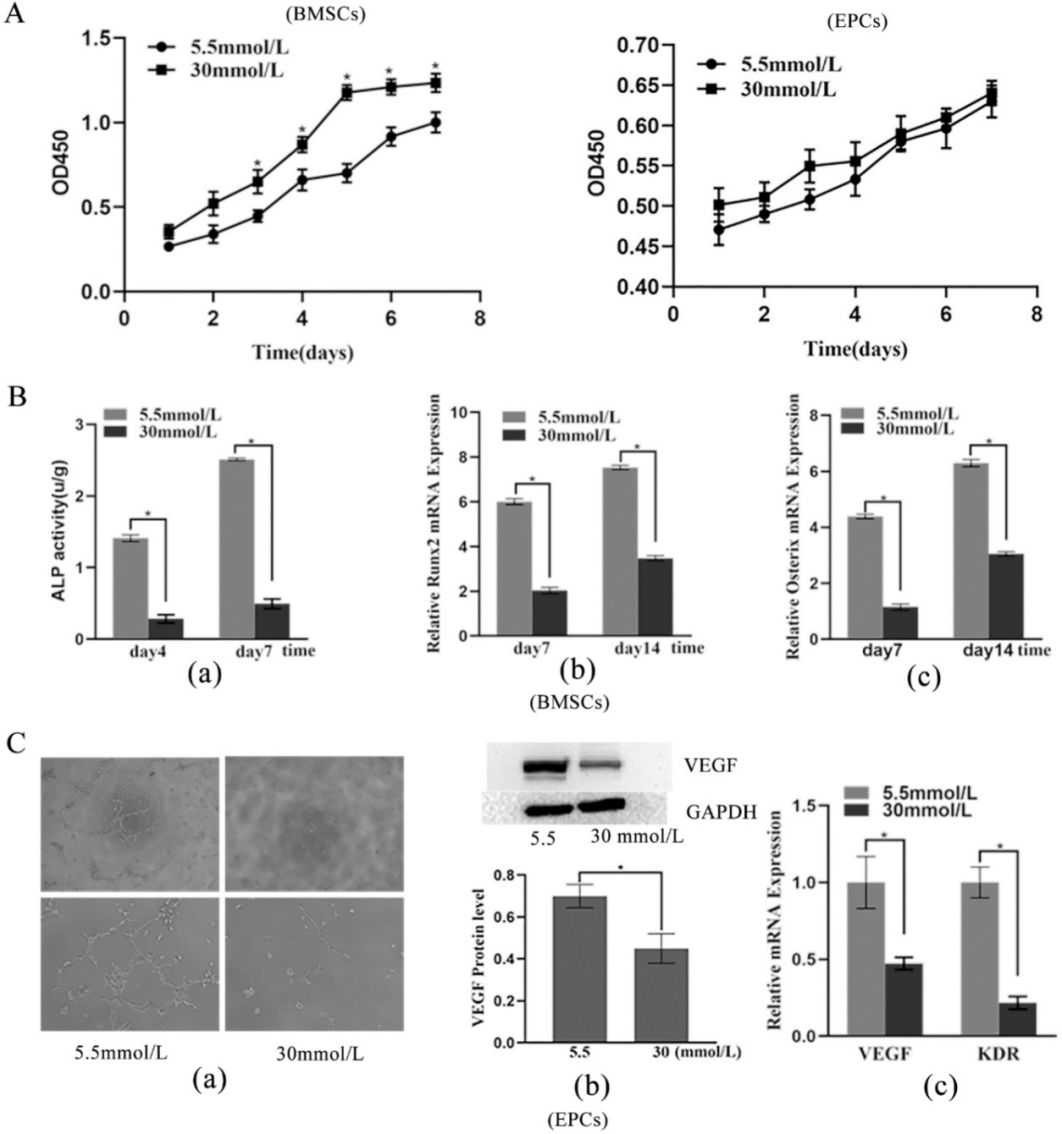
** Effects of high glucose on proliferation and differentiation of BMSCs and EPCs. A)** Cell viability of BMSCs and EPCs assessed by CCK-8 assay in different concentrations of glucose medium (*P<0.05); **B)** Effects of high glucose on differentiation of BMSCs. **a)** ALP ability of BMSCs (*P<0.05); **b)** Expressions of Runx2 mRNA (*P<0.05); **c)** Expressions of Osterix mRNA (*P<0.05). **C)** Effects of high glucose on differentiation of EPCs. **a)** Matrigel tubule formation experiment of EPCs in different concentrations of glucose medium (upper: ×40; lower: ×100); **b)** Relative protein expression of VEGF of EPCs in different concentrations of glucose medium (*P<0.05); **c)** Expressions of VEGF mRNA and KDR mRNA of EPCs in different concentrations of glucose medium (*P<0.05).

The CCK-8 assay results showed that cells treated with 30 mmol/L glucose had a markedly enhanced proliferative capacity starting on the third day (p < 0.05) (Figure 2A). ALP activity assays and RT-PCR were implemented to estimate the osteogenic capacity of BMSCs exposed to high glucose. The 30 mmol/L group showed weaker ALP activity than the 5.5 mmol/L group on the fourth and seventh days (p < 0.05) (Figure 2Ba). Moreover, the mRNA expression levels of Runx2 and Osterix were considerably downregulated in the 30 mmol/L group compared with the 5.5 mmol/L group (p < 0.05) (Figure 2Bb and Bc). In short, these results indicated that high-glucose promotes the proliferation but inhibits the differentiation of BMSCs.

### Effects of high glucose on EPC proliferation and differentiation

The CCK-8 assay results revealed that the 30 mmol/L group had an increased proliferative capacity, but the difference was not statistically significant (Figure 2A). The tubule formation assay showed that the 30 mmol/L group had a lower tubule formation capacity (Figure 2Ca). Moreover, the results showed that high glucose levels in the microenvironment inhibited the protein expression of VEGF and the mRNA expression of VEGF and KDR (p < 0.05) (Figure 2Cb and Cc), which indicated that high glucose inhibits the vascular formation ability of EPCs.

### Establishment of cell sheets and bilateral skull defects in T2DM rats

**Figure 3:**
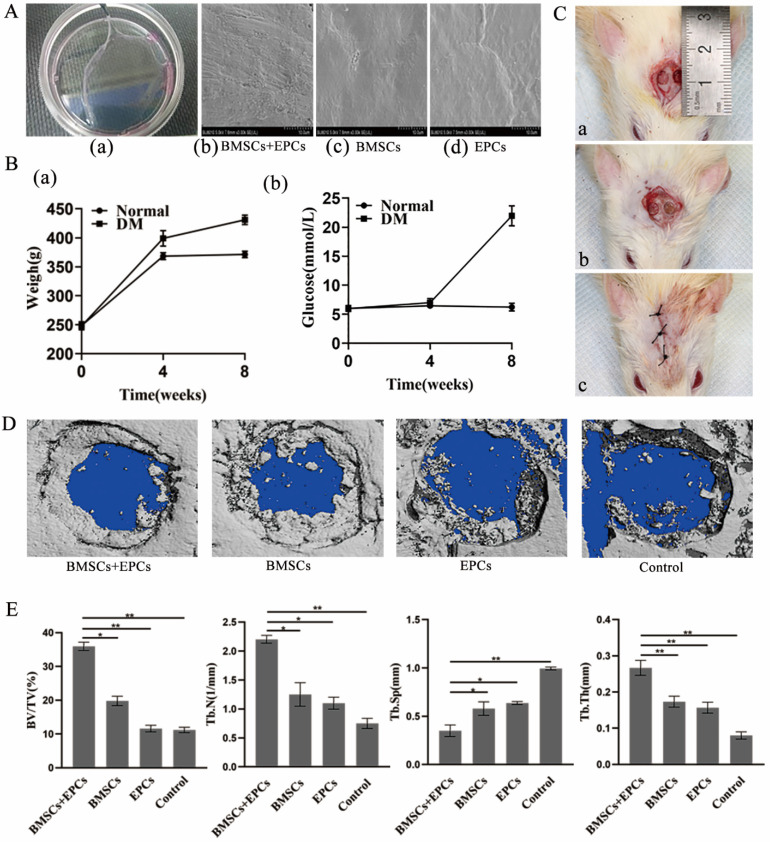
** Establishment of cell sheets and bilateral skull defect in T2DM rats and therapeutic effect of BMSCs-EPCs sheets on skull defects. A)** General observation of cell sheet and SEM observation of cell sheets (×3k). **B)** Establishment of T2DM rats. **a)** The changes in body weight between the two groups. **b)** The changes in glucose level between the two groups. **C)** Establishment of bilateral skull defect in T2DM rats **a)** construct 4 mm bilateral skull defects; **b)** implant the cell membrane into defects; **c)** suture. **D)** Micro-CT Scanning of calvarial CSD. **E)** Quantitative analysis of BV/TV, Tb.N, Tb.Sp, Tb.Th (*P<0.05, **P<0.001).

The cell sheets gradually separated from the edge of the dish and were easily detached with tweezers after 14 days of induction, which indicated the successful formation of cell sheets (Figure 3Aa). Scanning electron microscopy images showed cells embedded in extracellular matrix (Figure 3Ab-3Ad).

With the exception of one, all T2DM rats survived after anaesthesia without diabetic complications. Four weeks after inducing fat accumulation and insulin resistance, blood glucose levels were checked. Compared with that in the normal group, the glucose level of the T2DM group changed more significantly (P<0.05) (Figure 3Bb). After STZ injection, there was a significant difference in body weight between the T2DM group and normal group at 8 weeks (P<0.05) (Figure 3Ba). After anaesthesia, cutting and drilling, a 4 mm bilateral skull defect was generated (Figure 3Ca). Then, the cell membrane was implanted at the defect and sutured (Figure 3Cb-Cc).

### Therapeutic effect of BMSC-EPC sheets on skull defects in T2DM rats

**Figure 4:**
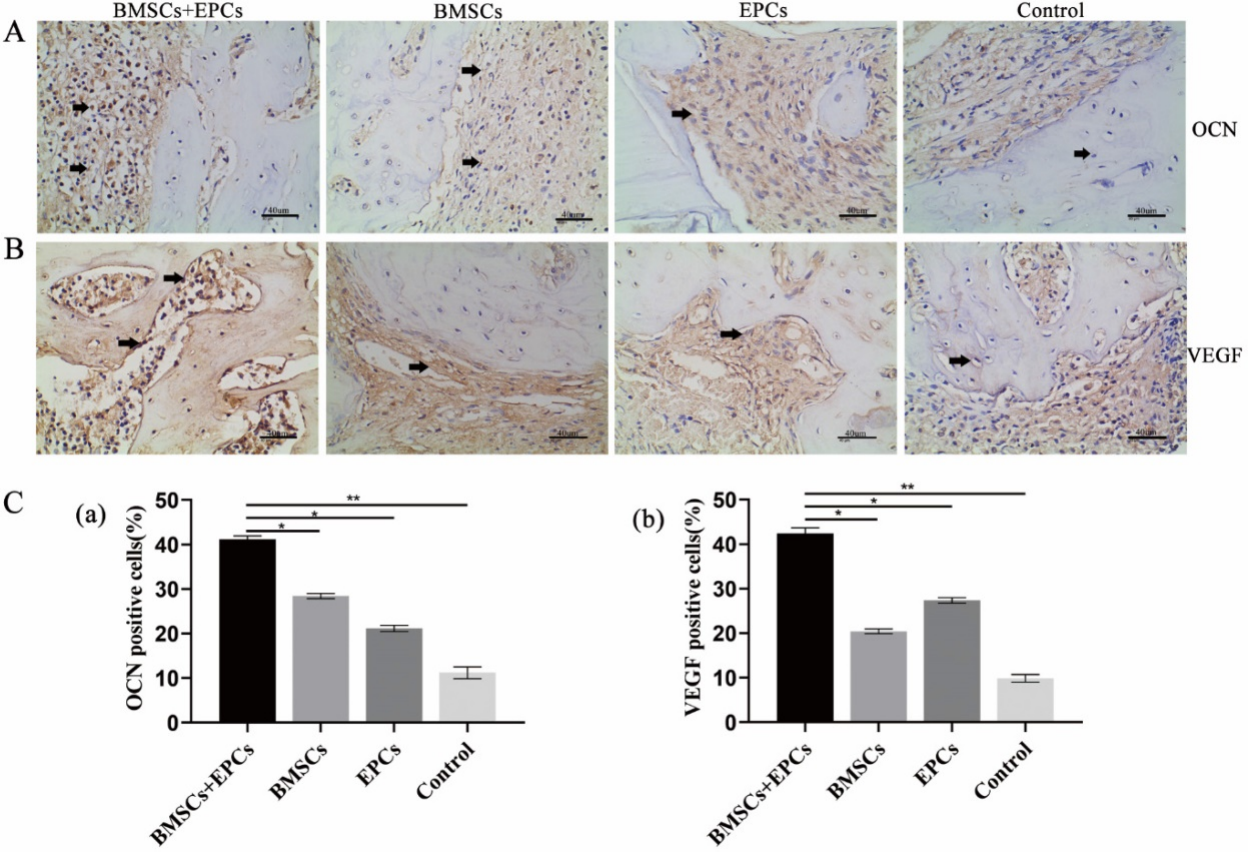
** Immunohistochemical of bone tissue sections. A)** Expression of bone marker proteins OCN in skull tissue sections from T2DM rats was detected by IHC analysis (black arrow: positive cells or intercellular substance; ×400). **B)** Expression of angiogenesis marker proteins VEGF in skull tissue sections from T2DM rats was detected by IHC analysis (black arrow: positive cells or intercellular substance; ×400). **C)** The analysis of OCN or VEGF positive cells (* P<0.05, **P<0.001).

Micro-CT images showed the formation of regenerated bone in the bone defect area of each group. Group A showed the strongest bone regeneration ability compared with the other groups at the time of sacrifice. In addition, there were notable differences in new bone formation in each group in the ROI. Statistical analysis of micro-CT parameters showed that group A had the highest BV/TV, Tb.N, and Tb.Th values and the lowest Tb.Sp value at 8 weeks. In addition, group A was notably different from the other groups in terms of BV/TV, Tb.N, Tb.Th and Tb.Sp (p < 0.05) (Figure 3D and 3E). Furthermore, the IHC analysis results demonstrated that the expression of OCN and VEGF in group A was notably higher than that in the other groups at 8 weeks (p < 0.05) (Figure 4). Thus, we concluded that BMSCs co-cultured with EPCs promote ossification *in vivo*.

### Effect of VEGF on the osteogenic differentiation of BMSCs

**Figure 5:**
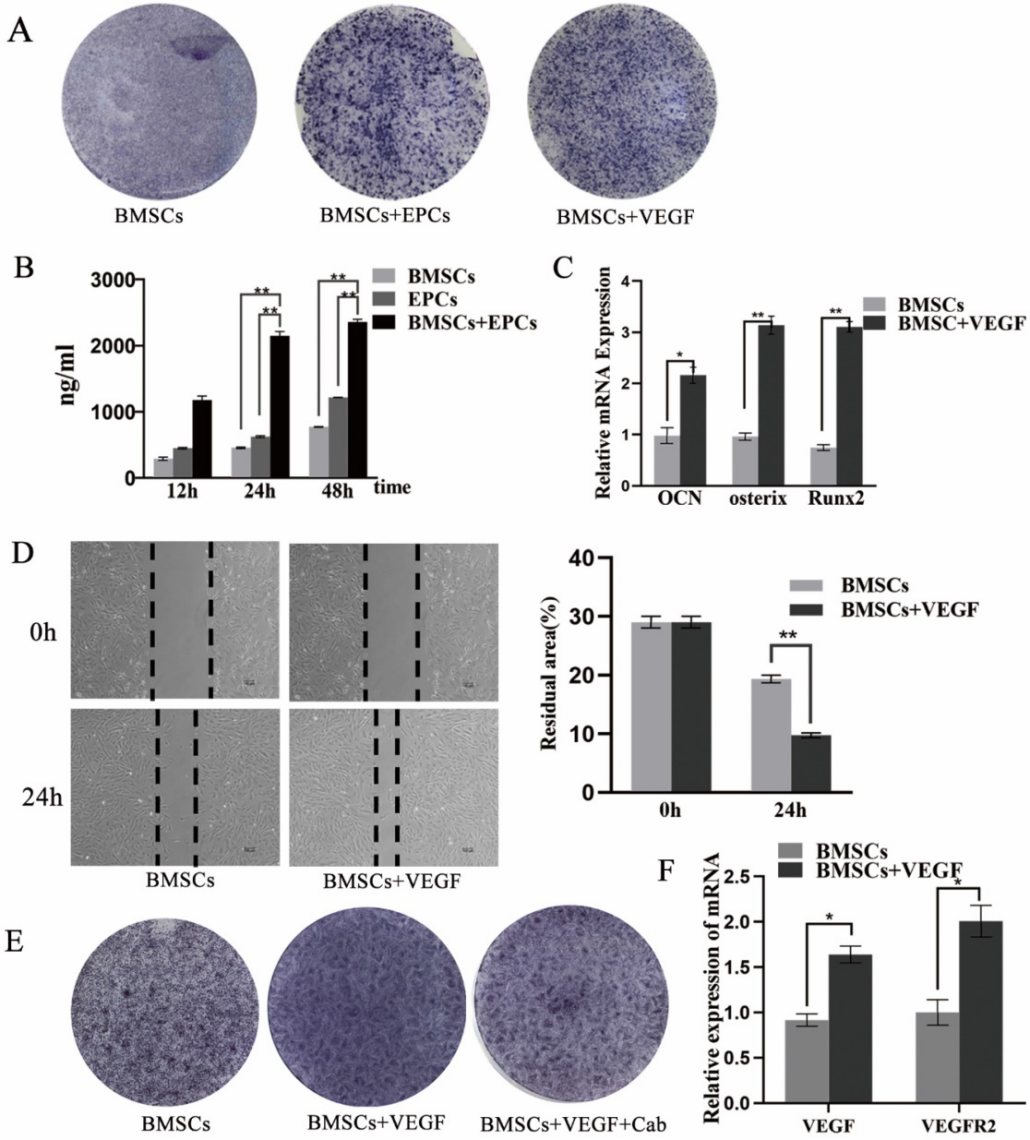
** VEGF can improve the differentiation and migration of BMSCs. A)** The ALP staining of BMSCs, BMSCs+ EPCs, or BMSCs+ VEGF; **B)** The level of VEGF in supernatant of EPCs with or without BMSCs assessed by elisa assay (**P<0.001); **C)** Expressions of OCN, Osterix and Runx2 mRNA of BMSCs with or without VEGF in high concentration of glucose (*P<0.05,** P<0.001); **D)** Scratch experiment of BMSCs with or without VEGF in high concentration of glucose (** P<0.001); **E)** The ALP staining of BMSCs, BMSCs+ VEGF, or BMSCs+ VEGF+Cab; **F)** Expressions of VEGF, and VEGFR2 mRNA of BMSCs with or without VEGF in high concentration of glucose (*P<0.05).

The results showed that the BMSCs/EPCs group presented a higher level of VEGF in the supernatant than the BMSCs group and the EPCs group (p < 0.05) (Figure 5B), which was consistent with the IHC results (Figure 4B). Moreover, the osteogenic capacity of BMSCs exposed to VEGF was evaluated by RT-PCR and ALP staining. The BMSCs exposed to VEGF displayed greater ALP activity than the control group on the seventh day (Figure 5A), and mRNA expression levels of OCN, Runx-2 and Osterix were considerably increased in the experimental group compared with the control group (p < 0.05) (Figure 5C). In addition, a scratch experiment was performed to explore changes in cell mobility after exposure to VEGF. The wound residual rate of BMSCs cultured with VEGF was 9.7±0.6%, which was significantly lower than that of the BMSC group (19.3±1.3%) after 24 h (p<0.001) (Figure 5D). All these data suggest that BMSCs co-cultured with EPCs could promote the osteogenic differentiation of BMSCs partially via VEGF.

Furthermore, the RT-PCR results showed that the mRNA expression levels of VEGF and VEGFR2 were considerably increased when BMSCs were exposed to VEGF (p < 0.05) (Figure 5F). IHC results also revealed that VEGF expression in bone cells was enhanced upon co-culture (Figure 4B). These results suggest that co-culture of BMSCs with EPCs not only promotes VEGF secretion but also increases endogenous VEGF levels. To verify whether VEGF acts on cells through VEGFR2, we added cabozantinib (Cab), a VEGFR2 inhibitor, to the culture medium during cell induction. The cells exposed to VEGF and Cab showed weaker ALP activity on the seventh day than those treated with VEGF only (Figure 5E).

**Figure 6:**
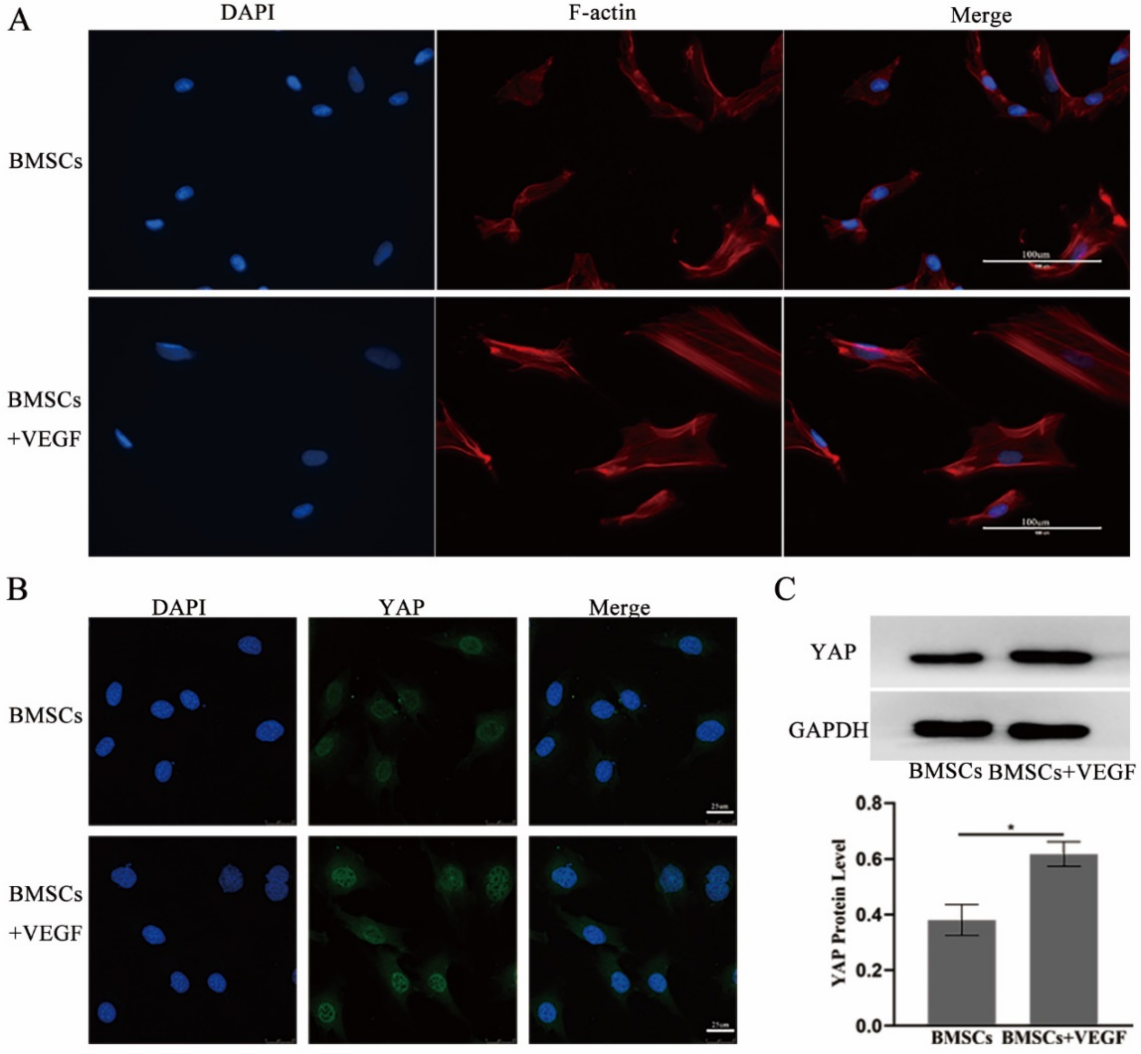
** VEGF improve the differentiation and migration of BMSCs may through F-actin/Yap pathway. A)** Immunofluorescence images of F-actin in BMSCs with or without VEGF (scale bar = 100 um). **B)** Immunofluorescence images of YAP in BMSCs with or without VEGF (scale bar = 25 um). **C)** YAP total protein expression detected by western blotting and quantitative analysis (*P<0.05).

To further explore the related mechanisms, we used immunofluorescence and western blotting to investigate the actin cytoskeleton changes and the total YAP expression and subcellular localization change when BMSCs were exposed to VEGF. The results showed that cells cultured with VEGF had a stronger actin cytoskeleton and a greater amount of nuclear and total YAP than the control group (Figure 6). Taken together, our results indicate that the F-actin/YAP pathways play an important role in the osteogenic differentiation of BMSCs.

## Discussion

It is widely believed that patients with T2DM have a high risk of fracture and bone healing damage 20, which is due to long-term exposure to a diabetic environment leading to changes in bone metabolism and impaired bone microarchitecture 3. BMSCs play an important role in tissue healing and regenerative medicine 21. Many different microenvironment conditions, such as high glucose, inflammation, and hypoxia, may change the physiological function of stem cells 22, 23. Recent studies have shown that the differentiation of skeletal stem cells is closely related to glucose metabolism 4. High glucose concentrations of 16.5 mM have been reported to interfere with the normal proliferation of rat bone marrow stem cells 24 and rat mandibular osteoblasts 25. Similarly, previous research found that high glucose levels in the microenvironment could inhibit the osteogenic differentiation of BMSCs, but exogenous transfer of the Runx2 gene could reverse this effect 16, which was consistent with our results.

Cardiovascular dysfunction is one of the major complications in diabetic patients. The role of EPCs in angiogenesis has been demonstrated, indicating that impaired EPC function in disease states may lead to vascular complications. Clinical observation showed that the number of circulating EPCs in diabetic patients was reduced and that their function was also impaired. *In vivo* studies of diabetic animals revealed 26 that EPC mobilization, differentiation, and tubule formation function were disrupted. Consistent with this, we found that high glucose levels in the microenvironment inhibited the vascularization of EPCs *in vitro*.

DM is linked with poor bone repair 27. Bone healing is closely related to the number and function of progenitor cells, and insufficient availability of MSCs may hinder the remodelling of injured tissue 28, 29. Moreover, fracture non-union is related to vascular insufficiencies at the fracture site 30. Recent studies have shown that EPCs have dynamic roles in maintaining MSC stemness and in regulating MSC differentiation potential 31. Furthermore, EPCs could improve the osteogenic differentiation of BMSCs and enhance ectopic bone formation 11, and combined transplantation of EPCs and MSCs improves the healing of bone defects 32. Given this evidence, EPCs may be implicated in restoring BMSC function under diabetic conditions. To verify our hypothesis, we detected the effect of EPCs on the function of BMSCs exposed to high glucose and successfully generated bilateral skull defects in T2DM rats and implanted cell sheets into them. The results indicated that EPCs could enhance the osteogenic differentiation of BMSCs *in vitro* and *in vivo*. When transplanted into T2DM rats, the EPC-BMSC sheets facilitated stronger bone regeneration than the other sheets and the control. However, there were some differences between the model defects and clinical defects. We used cell sheets in the skull bone defect model, which has defects of only 4 mm where mechanical stability still exists, but in larger defects, the use of cell sheets alone does not provide mechanical stability. The therapeutic potential of EPC-BMSC cell sheets combined with bone transplantation, intraosseous implants and other tissue engineering methods in bone defects should be further explored.

The expression of specific growth factors during healing, such as TGFs, BMPs, VEGF, FGFs and PDGFs, suggests that these secretory factors may play a role in bone repair 8, 33. Among them, VEGF has attracted special interest because of its ability to induce angiogenesis 13. Several studies have shown that the expression of angiogenic genes (VEGF-A and VEGF-C) and proteins in MSCs isolated from diabetic patients is decreased 34, 35. Several reports found that endogenous VEGF and the delivery of exogenous VEGF enhanced bone formation and bone healing by improving angiogenesis 14. We demonstrated that there was a higher level of VEGF in the supernatant of BMSCs with EPCs, and we also demonstrated that VEGF could promote BMSC osteogenic differentiation and migration *in vitro*. Moreover, higher expression of VEGF was found in the BMSC-EPC group, as indicated by IHC *in vivo*, and the mRNA expression of VEGF was considerably increased when BMSCs were exposed to VEGF with RT-PCR *in vitro*. This could be one of the reasons that EPC-BMSC sheets performed better than others in our research. Further research on the mechanisms by which BMSCs co-cultured with EPCs enhance bone defect healing should be explored.

VEGF acts on cells through a variety of receptors, including VEGFR1, VEGFR2 and VEGFR3, among which VEGFR2 is the most effective and extensive mediator. The results showed that VEGF could enhance the osteogenic differentiation of BMSCs, and the effect was reversed when VEGFR2 receptor inhibitors were added. In addition, the RT-PCR results showed that the mRNA expression level of VEGFR2 increased after exposure to VEGF, which suggested that VEGFR2 is the main medium by which VEGF enters BMSCs. YAP and TAZ are transcriptional co-activators. Under certain conditions, YAP/TAZ can be transferred from the cytoplasm to the nucleus to regulate gene expression. Studies have shown that in MSCs and osteoblasts, YAP/TAZ can promote osteogenic differentiation by regulating the transcription factor Runx2 36-39. The results of this experiment prove that VEGF can regulate YAP/TAZ and facilitate YAP entry into the nucleus. Other scholars believe that by regulating cytoskeletal tension, YAP/TAZ nuclear transfer is promoted, and Runx2 and PPARγ combine in the nucleus to participate in the determination of BMSC osteogenic or adipogenic differentiation 40. YAP/TAZ has been shown to be regulated by the actin cytoskeleton 41. When blocking F-actin or inhibiting the Rho family, YAP/TAZ loses activity 41-43. In our study, we found that VEGF stabilized the F-actin bands of BMSCs. In conclusion, we conclude that VEGF may promote YAP nuclear localization through robust F-actin bands, thereby facilitating BMSC osteogenesis. However, the specific roles of the YAP/TAZ pathway and the change in cytoskeletal tension in this process need to be further clarified.

## Figures and Tables

**Table 1:** Primers used for qRT-PCR

Gene	Forward primer sequence (5′-3′)	Reverse primer sequence (5′-3′)
Runx2	CCGAGACCAACCGAGTCATTTA	AAGAGGCTGTTTGACGCCAT
Osterix	GCCAGTAATCTTCGTGCCAG	TAGTGAGCTTCTTCCTGGGGA
OCN	CTCAACAATGGACTTGGAGCC	GGCAACACATGCCCTAAACG
VEGF	TTTTGCTTCCTATTCCCCTCTT	CTCCTGCTACCTCTTTCCTCTG
KDR	CCCAGAGTGGTTGGAAATGATA	TAGACATAAACGATGGAGGAGAC
β-actin	CCCGCGAGTACAACCTTCTTG	GTCATCCATGGCGAACTGGTG
GAPDH	AGTGCCAGCCTCGTCTCATA	GATGGTGATGGGTTTCCCGT
