# Co-culture with Endothelial Progenitor Cells promotes the Osteogenesis of Bone Mesenchymal Stem Cells via the VEGF-YAP axis in high-glucose environments: Erratum

**DOI:** 10.7150/ijms.73905

**Published:** 2022-06-12

**Authors:** Peilian Wu, Xia Zhang, Yun Hu, Dongrong Liu, Jinlin Song, Wenjie Xu, Hao Tan, Rui Lu, Leilei Zheng

**Affiliations:** 1 The Affiliated Stomatology Hospital, Chongqing Medical University, Chongqing, 401147, China.; 2 Chongqing Key Laboratory of Oral Diseases and Biomedical Sciences, Chongqing, 401147, China.; 3 Chongqing Municipal Key Laboratory of Oral Biomedical Engineering of Higher Education, Chongqing, 401147, China.; 4 West china dental hospital of Chongqing, Chongqing, 401147, China.

In our paper, Figure 1B-b and Figure 1 should be corrected as follows.

## Figures and Tables

**Figure A:**
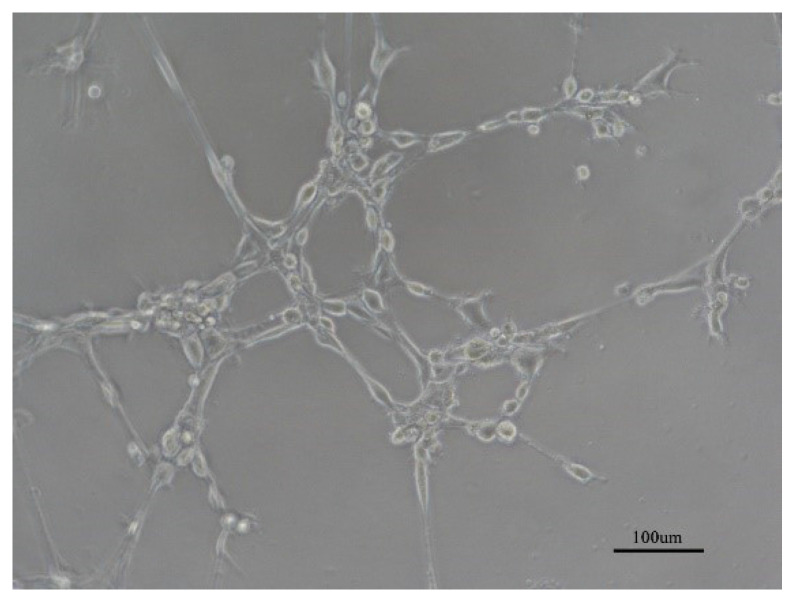
Corrected Figure 1B-b.

**Figure B:**
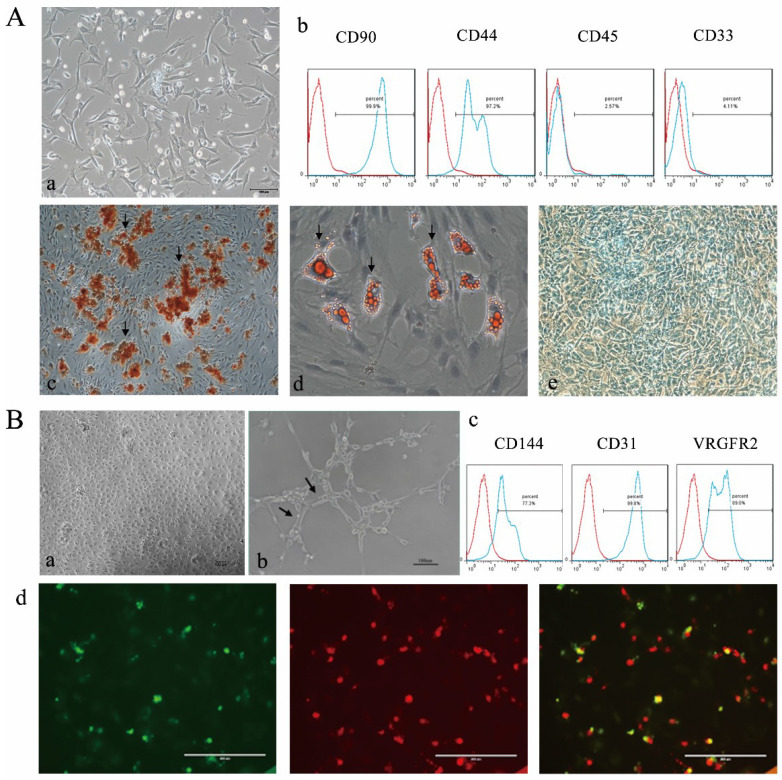
Corrected Figure 1. Characterization of BMSCs and EPCs. A) Isolation and differentiate of BMSCs a morphology of BNSCs,P0×100; b) Cell surface markers of BMSCs c) Alizarin red staining of BMSCs after osteogenic induction (×100; Black arrows indicate calcium deposits); d) Oil red O staining of BMSCs after adipogenic induction (×200; The black arrow indicates lipid droplets); e Alcain staining of BMSCs after chrondrogenic induction (×100). B) Isolation and identification EPCs a) morphology of epc, P1×100; b) Matrigel tubule formation experiment of EPCs (×100.Black arrow indicates tubule). c) Cell surface markers of EPCs d) Double fluorescence staining experiment of EPCs, (×200.green: FITC-UEA-1 red: DiI-Ac-LDL, yellow: fluorescence coincidence).

